# Etymologia: Cephalosporin

**DOI:** 10.3201/eid1108.ET1108

**Published:** 2005-08

**Keywords:** etymology, Etymologia, Cephalosporin

## [sef′′ə-lo-spor′in]

Any of a class of broad-spectrum, relatively penicillinase-resistant, ®-lactam antimicrobial drugs originally derived from species of the fungus *Acremonium *(formerly called *Cephalosporium*). Italian scientist Giuseppe Brotzu first isolated the parent compound cephalosporin C from a sewer in Sardinia in 1948. Cephalosporins available for medical use today are semisynthetic derivatives of this natural antimicrobial compound.

**Sources: **Dorland's illustrated medical dictionary. 30th ed. Philadelphia: Saunders; 2003. and Merriam-Webster's collegiate dictionary. 11th ed. Springfield (MA): Merriam-Webster's, Inc; 2003.

